# Early Changes in Blood Urea Nitrogen (BUN) Can Predict Mortality in Acute Pancreatitis: Comparative Study between BISAP Score, APACHE-II, and Other Laboratory Markers—A Prospective Observational Study

**DOI:** 10.1155/2021/6643595

**Published:** 2021-03-22

**Authors:** Elizabeth Pando, Piero Alberti, Rodrigo Mata, María José Gomez, Laura Vidal, Arturo Cirera, Cristina Dopazo, Laia Blanco, Concepción Gomez, Mireia Caralt, Joaquim Balsells, Ramón Charco

**Affiliations:** Department of Hepato-Pancreato-Biliary and Transplant Surgery, Hospital Universitari Vall D'Hebron, Universitat Autònoma de Barcelona, Barcelona, Spain

## Abstract

**Background:**

Changes in BUN have been proposed as a risk factor for complications in acute pancreatitis (AP). Our study aimed to compare changes in BUN versus the Bedside Index for Severity in Acute Pancreatitis (BISAP) score and the Acute Physiology and Chronic Health Evaluation-II score (APACHE-II), as well as other laboratory tests such as haematocrit and its variations over 24 h and C-reactive protein, in order to determine the most accurate test for predicting mortality and severity outcomes in AP.

**Methods:**

Clinical data of 410 AP patients, prospectively enrolled for study at our institution, were analyzed. We define AP according to Atlanta classification (AC) 2012. The laboratory test's predictive accuracy was measured using area-under-the-curve receiver-operating characteristics (AUC) analysis and sensitivity and specificity tests.

**Results:**

Rise in BUN was the only score related to mortality on the multivariate analysis (*p*=0.000, OR: 12.7; CI 95%: 4.2−16.6). On the comparative analysis of AUC, the rise in BUN was an accurate test in predicting mortality (AUC: 0.842) and persisting multiorgan failure (AUC: 0.828), similar to the BISAP score (AUC: 0.836 and 0.850) and APACHE-II (AUC: 0.756 and 0.741). The BISAP score outperformed both APACHE-II and rise in BUN at 24 hours in predicting severe AP (AUC: 0.873 vs. 0.761 and 0.756, respectively).

**Conclusion:**

Rise in BUN at 24 hours is a quick and reliable test in predicting mortality and persisting multiorgan failure in AP patients.

## 1. Introduction

Severe acute pancreatitis (SAP) occurs in nearly 20% of acute pancreatitis (AP) patients, and it is related to higher mortality rates around 30% [1, 2]. The mortality is significantly higher in patients with necrotizing pancreatitis (NP) due to the incidence of systemic and local complications, including infection of the pancreatic necrosis in 20% of patients [2–4], and recently, our group has demonstrated that extrapancreatic infection played a role in predicting the severity and local complications in AP [5].

Mortality and severity prediction of AP remains to be a challenge. A laboratory test, easy to perform, and with good predictive power is necessary, contrary to classical invasive laboratory tests, such as APACHE-II [6–8].

Recently, the blood urea nitrogen (BUN) changes have gained interest due to its high sensibility in predicting the severity of SAP and mortality [9–13], as a rapid and straightforward test admission. However, few studies have compared it with one of the essential clinical scores such as BISAP and APACHE-II, with contradictory results [7, 9, 12–15].

The rise in BUN (azotemia) in AP patients are explained by a mechanism of acute renal injury consequence of (a) the loss of intravascular volume, due to interstitial extravasations related to the systemic inflammatory response (SIRS) [9, 12, 16] and (b) a direct renal injury mechanism, occurring in AP promoted by the releasing of activated enzymes such trypsin and chymotrypsin, inflammatory mediators, and cytokines (TNF-alpha, IL-8, IL-6, and IL-1 beta) [17].

Our study aimed to explore the role of BUN changes, compared with APACHE-II, haemoconcentration, and C-reactive protein (CRP), in predicting mortality and severity in patients diagnosed with acute pancreatitis.

## 2. Methods

### 2.1. Study Design

A prospectively single-cohort observational study of adult patients diagnosed with acute pancreatitis in a third level referral centre was designed in order to evaluate the role of BUN in mortality and severity predictor in patients with AP.

Inclusion criteria were patients with diagnostic of AP according to the 2012 Atlanta classification [4]. Exclusion criteria were patients with renal chronic disease patients at stages III-IV, patients with more than 96 hours of an interval of time between the onset of pain and admission, and transfer patients from other hospitals.

### 2.2. Serum Variables, BISAP, and APACHE-II Scores

BUN, haematocrit, and C-reactive protein (CRP) were determined at admission, 24 hours, and 48 hours after admission. Any increase in the value of BUN or haematocrit after 24 hours was considered as positive for a “rising” criteria. APACHE-II and BISAP score were determined at admission only [6, 12, 18–20].

### 2.3. Acute Pancreatitis Definitions

Acute pancreatitis severity was based on the 2012 Atlanta classification [4] as (1) mild acute pancreatitis, requires no organ failure or local or systemic complication; (2) moderately severe acute pancreatitis, requires an organ failure (OF) to be resolved within 48 h (transient organ failure and local or systemic complications without organ failure) and local or systemic complications without persistent organ failure (POF); finally, (3) severe acute pancreatitis, persistent (>48 h) single or persistent multiple organ failure (PMOF). Organ failure was defined as a score of 2 or more for one of three organs (renal, cardiovascular, or respiratory) using the modified Marshall scoring system [21].

### 2.4. Acute Pancreatitis Management

According to the international guidelines [22], our institutional management protocol [5] of AP included initially fluid therapy according to the patient characteristics with a goal of a urinary output of more than 0.5 ml/kg/hour, based on ringer lactate and isotonic sodium chloride solutions. When severe AP was suspected, the patient was referred to the ICU team for management and counselling.

### 2.5. Pancreatic Necrosis

Definition for pancreatic necrosis was nonenhancement in pancreatic tissue after CT-contrast and also the presence of extrapancreatic fat necrosis. The CT protocol for pancreatic evaluation consists of a retarded venous phase after 35 seconds of venous contrast administration. CT scan was performed after at least 72 hours from onset of pain. Local complications were defined according to Atlanta classifications [4].

Infected pancreatic necrosis (IPN) was defined by a positive culture after surgical, radiological, or endoscopic approach.

Suspected IPN was defined in a patient with sepsis without an extrapancreatic origin of infection (catheter line infection, respiratory tract infection, and urinary tract infection), with or without finding of gas bubbles on CT scan (in patients without previous interventional procedures against digestive tract) with worsening of the clinical status. We do not use antibiotic prophylaxis for pancreatic necrosis. In the case of IPN, a step-up approach policy was carried out.

Mortality was defined as a fatality event during admission or up to 90 days after discharge.

### 2.6. Statistical Analysis

Univariate and multivariate analyses were performed to explore the association of every score with significant outcomes. The cutoff values corresponded to the highest value of the Youden index of every score were analyzed. The predictive accuracy of the laboratory test and scores was measured using area-under-the-curve (AUC) receiver-operating characteristics (ROC) analysis and sensitivity and specificity tests with 95% confidence interval (CI). Chi-square or Fisher's exact tests were used for comparing qualitative variables. A comparison of the AUC was performed using Delong's test. Statistical significance was considered when the *p* value of <0.05 was achieved.

### 2.7. Ethics

The study was conducted in compliance with the principles of the Declaration of Helsinki and Good Clinical Practice guidelines. The Hospital Institutional Review Board approved this study with code PR (AG) 316/2016.

## 3. Results

From November 2015 to January 2020, a total of 459 patients met the inclusion criteria. From these, 49 patients were excluded, eighteen were transferred patients, and thirty-one arrived at the emergency department more than 96 hours after onset of abdominal pain. Finally, 410 patients were analyzed. The basal characteristics are given in Table 1.

The optimal cutoff values to consider a positive score were obtained according to the Youden index and were as follows: rise in haematocrit at 24 hours, a variation >0.6%; rise in BUN at 24 hours, a variation >1.87 mg/dl; APACHE-II, >8 points; haematocrit at admission, >45%; BUN at admission, >23.3 mg/dl; and BISAP score, ≥2 points. The cutoff values for highest sensitivity and specificity were the same for mortality, persistent multiorgan failure, and SAP.  Table 2 describes the incidences of major outcomes according to a positive or negative score.

On the univariate analysis, all the major outcomes analyzed were more frequent in the group of positive scores versus negative scores: rise in haematocrit at 24 hours, rise in BUN at 24 hours, APACHE-II, BISAP, haematocrit at admission, and BUN at admission, with the only exception of C-reactive protein at admission. C-reactive protein did not show an association with the major outcomes analyzed.

BISAP ≥2 and rise in BUN at 24 hours showed the highest OR values on the univariate analysis for major outcomes. Incidence of mortality for a BISAP ≥2 was 12% versus 0.4% in the BISAP <2 group (*p*=0.000, OR: 30.5; CI 95%: 4.0–228.9), and for a positive rise in BUN at 24 hours, we found a 27.7% of incidence of mortality compared with 1.4% with no rise in BUN at the 24 hours group (*p*=0.000, OR: 26.0; CI 95%: 9.2–73.4). The incidence of multiorgan failure for BISAP >2 was 16.3% versus 0.4% in the BISAP ≤2 group (*p*=0.000, OR: 43.8; CI 95%: 5.9–324.6), and positive rise in BUN at 24 hours was 33.8% versus 2.6% compared with no rising in BUN at 24 hours (*p*=0.000, OR: 19.1; CI 95%: 8.2–44.1). Similarly, the odds ratio of severe AP for BISAP >2 and rising in BUN at 24 hours were higher than the other scores analyzed (Table 2).

In the multivariate analysis, rise in BUN at 24 hours was the only score related significantly with mortality (*p*=0.000, OR: 12.7; CI 95%: 4.1–38.2). For multiorgan persistent failure, we found that most of the scores had a significant association: rise in haematocrit at 24 hours (*p*=0.001, OR 7.5; CI 95%: 2.3–24.2), haematocrit at admission > 45% (*p*=0.005, OR: 4.1; CI 95%: 1.5–11.5), being higher for rising in BUN at 24 hours (*p*=0.000, OR: 9.8; CI 95%: 3.8–25.3), and for BISAP > 2 (*p*=0.017, OR: 13.5; CI 95%: 1.5–115.2). Similarly, the multivariate analysis for severe AP showed a significant association with rising for BUN at admission >23.3 mg/dl (*p*=0.012, OR: 2.9; CI 95%: 1.2–6.9), rise in haematocrit at 24 hours (*p*=0.033, OR: 2.9; CI 95%: 1.09–8.1), rising in BUN at 24 hours (*p*=0.000, OR: 5.1; CI 95% 2.3–11.1), and BISAP > 2 (*p*=0.003, OR: 23; CI 95%: 2.8–182).

BISAP score showed the highest sensitivity (95.6%) but low specificity (58.1%) in predicting mortality. However, the best value of the Youden index was for rise in BUN at 24 hours (*J* = 0.661) with a sensitivity of 78.2% and specificity of 87.6%, being higher than APACHE-II (69.5% and 73.1% for sensitivity and specificity, respectively) and BUN at admission >23.3 mg/dl (sensitivity: 65.2%; specificity 71.8%).

For persistent multiorgan failure, the BISAP score showed a higher sensitivity (96.7%) but low specificity (59.3%). Rise in BUN showed slight advantage for both sensitivity (70.9%) and specificity (88.6%) compared to APACHE (sensitivity 67.7%; specificity: 73.8%) or BUN at admission (sensitivity 70.9%; specificity 68.3%).

However, regarding severe AP prediction, according to Atlanta 2012, the BISAP score showed superiority in sensitivity (97.7%) compared with APACHE-II and rise in BUN at 24 h (sensitivity of 82.2% and 55.5%, respectively).

**Figure 1:**
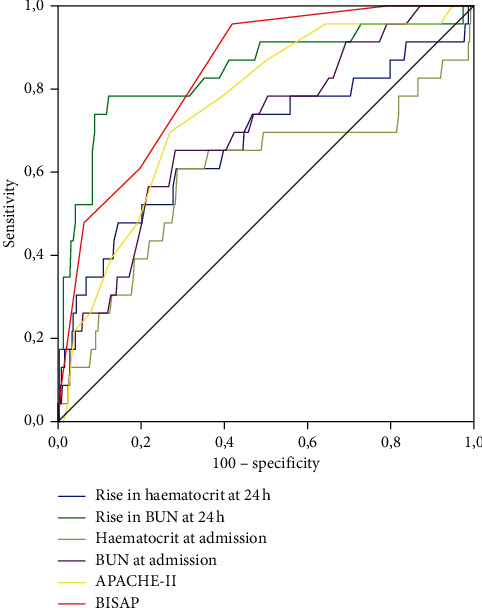
Comparison of ROC curves for mortality.

In the AUC analysis, we found that rise in BUN at 24 hours showed the highest value of AUC (0.842) in predicting mortality, exceeding all scores examined, including BISAP (AUC: 0.836), APACHE-II (AUC: 0.756), BUN at admission (AUC: 0.698), and haematocrit variations (Table 3). However, when comparing the AUC using the De Long test, no differences were found between BISAP, APACHE-II, and BUN levels at admission (Figure 1). In the AUC analysis for persistent multiorgan failure, BISAP score and rise in BUN at 24 hours showed the highest AUC value (0.850 and 0.828) followed by APACHE-II (0.741), and no significant differences between curves in the De Long test analysis.

Using the De Long test, the comparison of AUCs for SAP showed that the BISAP score had significant superiority over APACHE-II and the rise in BUN at 24 h (AUC: 0.873 vs. 0.761 vs. 0.756, respectively). There was no role for haematocrit at admission, haemoconcentration variations, and CRP ≥15 mg/dl in predicting mortality, persistent organ failure, or persistent single organ failure or SAP in the AUC analysis.

## 4. Discussion

Our study found that a rise in BUN at 24 hours (a variation > 1.87 mg/dl from admission) had good sensitivity and specificity and AUC values for mortality and persistent multiorgan failure in AP similar to the BISAP score and APACHE-II. Additionally, its occurrence was related to higher mortality, multiorgan failure, and severe AP rates at the multivariate analysis.

To date, few reports had compared the BUN value and its variations with the BISAP score or APACHE-II, the most potent scores predicting mortality in acute pancreatitis [9, 12, 14, 15]. Therefore, our study adds new data supporting that rise in BUN at 24 hours is a reliable score, with reasonable accuracy in predicting mortality and severity in AP. Additionally, this is the only study in demonstrating that the rise in BUN at 24 hours has a superior sensitivity and specificity in predicting mortality compared with one of the most potent scores so far, the APACHE-II.

Wu Bu et al [13] found that the rise in BUN at 24 hours predicted mortality in a similar way to APACHE-II ≥ 8 (AUC 0.84 vs. 0.80, respectively). Few studies compared rise in BUN at 24 hours with APACHE-II ≥8 or BISAP score and showed contradictory conclusions or did not explore its relation with mortality [7, 9, 12, 14]. Koutroumpakis et al. reported the collected data from high volume centres. They found that BUN values over 20 mg/dl at admission predicts POF or severe AP and designs an algorithm based on the rise in BUN and haemoconcentration associated with higher mortality rates [12].

He W et al. identified that the rise in BUN at 48 hours showed a better prediction for mortality, outperforming the APACHE-II at 48 hours and BISAP, among other scores. However, in their analysis, rise in BUN on admission or at 24 hours was not superior to the APACHE-II ≥8 in predicting mortality [9]. Vasudevan et al. found that BUN levels >17 mg/dl on admission was superior to the APACHE-II and BISAP in predicting mortality; however, they did not explore the BUN variations at 24 or 48 hours [14].

In another study comparing different scores, including BUN >40 mg/dl on admission, APACHE-II ≥8, and the presence of inflammation beyond the rectovesical space on computed tomography, the latter obtained the highest specificity for predicting mortality [15].

Interestingly, the haemoconcentration or rise in haematocrit was not related to mortality and both were outperformed by the rise in BUN at 24 hours and the BISAP score. These results are contradictory to previous reports [12, 23].

Changes in BUN reflect a broad spectrum of essential events in AP, including haemoconcentration, microcirculatory changes, and renal impairment, as well as an increased protein catabolism. As we know, all of these are frequently associated with severe AP [24–28]. Additionally, rise in BUN could be a consequence of inappropriate fluid reposition in AP patients or fluid sequestration mechanism, being this, a frequent phenomenon in SAP due to SIRS and the release of cytokines that promotes vasodilatation and distributive shock [29, 30].

The rise in BUN is also a reflection of acute kidney injury (AKI) in patients with AP [17]. In our cohort, creatinine levels correlated positively with BUN levels at admission (*r*^2^ = 0.72). The explanation for AKI in AP evolves diverse phenomena, including volume depletion due to extravasation of fluids from the vascular space, favoured by the release of inflammatory, vascular and humoral mediators, and direct glomerular injury mediated by cytokines-activated enzymes, proteases, free radicals, and others. Interestingly, fluid overload also mediates AKI in AP and in the critical care scenario [31]. In AP patients, one explanation relies on the fact that hyperfluid reposition could predispose to compartment syndrome, diminishing renal perfusion with a consequent function impairment.

Additionally, hypervolaemia may stretch the vascular wall and worsen vascular permeability. Simultaneously, fluid overload could be a consequence of AKI, explained by the endothelial dysfunction secondary to inflammation or ischemia/toxic injury, favouring capillary leakage [32]. Consequently, the increasing azotemia (rise in BUN) in AP patients reflects renal function impairment, which could lead to or be the consequence of a fluid overload due to endothelial leakage favoured by hyperfluid reposition therapies.

It was thought that hyperfluid hydration policy in AP patients could be a better strategy in the past [33]. Regarding hyperfluid reposition in AP patients, contradictory findings have been published on the incidence of organ failure and abdominal compartment syndrome when hyperfluid resuscitation policy was installed [34, 35]. Recent data showed that hyperfluid hydration in AP increases the risk of acute kidney injury, pulmonary oedema, compartmental syndrome, sepsis, and mortality [36–42]. In this line, some studies recently explored the impact of fluid sequestration in AP, showing that its presence is related to poor outcomes, including severe AP persistent organ failure, ICU admission, or more hospital stay [43, 44].

In consequence, if AKI is suspected, hyperfluid hydration will worsen the clinical scenario. Unfortunately, the tools to guide fluid management to establish when fluid administration is no longer beneficial are inadequate. In the past, the fluid reposition based on haemoconcentration was proposed, without robust data supporting this strategy; more recently, new publications reported worse outcomes [45].

According to this, recent reports, using advanced machine-learning techniques, identified that rise in BUN concentration was a risk factor for nonvolume responsiveness in patients with suspected acute kidney injury [46]. Our study supports that the rise in BUN is a potent risk factor for mortality. Interestingly, we found a strong relationship between indirect signs of fluid overload such as pleural effusion and BUN variations, being higher for a rise in BUN at 24 hours (OR: 3.3; CI 95%: 1.8–5.9) than BUN at admission (OR: 2.6; CI 95%: 1.6–4.1). These results favour the hypothesis that fluid overload is an evolutive process and could influence BUN values.

Nevertheless, new markers are needed to identify patients who will benefit from aggressive or restrictive fluid therapy reposition. B-type natriuretic peptide (BNP) could be a potential marker in this scenario. BNP is a cardiac biomarker secreted by the ventricular myocytes in response to ventricular wall stress secondary to different causes as fluid overload. BNP-guided treatment strategy for fluid reposition had been explored in other scenarios (cardiac insufficiency, critical care patients), obtaining better clinical outcomes when compared with standard treatments [47, 48]. According to the BNP values, a strategy of fluid restriction or fluid reposition will diminish the risk of fluid overload, especially in AP patients with suspected AKI.

Based on our findings, a reduction in BUN after 24 hours should be proposed as a goal for an appropriate fluid reposition in these patients in a well-designed prospective study.

Our institution had developed a research field looking for new prognostic factors in acute pancreatitis developing a dedicated prospective register of AP patients. One of our study's strengths is the fact that we only included patients from our institution, with less than 96 hours of symptoms at admission, and transferred patients were excluded. We believe these exclusion criteria could diminish the bias regarding the time when intravascular changes occur and when its treatment is applied. All our definitions were based on the current Atlanta 2012 classification criteria [4]; this is a crucial point to address due to the variability in criteria definitions of AP severity used in previous studies [13, 24, 49].

Our study has some limitations; one of these is that we did not make a timeline determination of the APACHE-II and BUN more than 48 h or 72 hours. We believe an “ideal” score needs to be fast, noninvasive, straightforward, and preferably early at the disease.

Additionally, for rising in BUN and haematocrit, any upward variation in the BUN was considered; even in the case, this variation will not exceed typical values.

Not all patients underwent an abdominal CT; given this, we did not analyze the predictive value of the scores and laboratory markers studied, regarding pancreatic necrosis occurrence.

Our study is one of the few studies on the literature that compares BUN variations with widely validated scores such as the APACHE-II and BISAP. We demonstrate the similarity of the rise in BUN at 24 h in predicted mortality, compared with BISAP or APACHE-II. The advantage of using the rise in BUN relies on the fact that it is simple, less invasive, and quickly tested, with broad availability in emergency surgical departments.

Future clinical trials need to be focused on the reduction in BUN after 24 hours as an endpoint in order to prevent AP severity and mortality.

## 5. Conclusions

A rise in BUN at 24 hours predicts mortality, similar to the most reliable scores, such as APACHE-II and BISAP. Monitoring BUN could help identify patients at risk of mortality with a fast and available test. A rise in BUN at 24 hours is a risk factor for significant outcomes such as mortality, multiorgan failure, and severe AP.

## Figures and Tables

**Table 1:** Basal population characteristics.

Variable	*n* = 410
Age, median ± SD	65.4 ± 18.6
Gender, male, *n* (%)	209 (51)
ASA score, *n* (%)	
I	82 (20)
II	182 (44.4)
III	144 (35.1)
IV	2 (0.5)
BMI, median ± SD	28.4 ± 5.2
Comorbidities	
Diabetes mellitus, *n* (%)	89 (21.7)
Dyslipidemia, *n* (%)	131 (32)
Cardiovascular disease, *n* (%)	105 (25.6)
Higher blood pressure, *n* (%)	222 (54.1)
Renal chronic disease, *n* (%)	30 (7.3)
Respiratory chronic disease, *n* (%)	59 (14.4)
AP aetiology, *n* (%)	
Biliary	319 (77.8)
Alcohol	40 (9.8)
Idiopathic	17 (4.1)
Post-ERCP	13 (3.2)
Hypertriglyceridemia	3 (0.7)
Others	18 (4.3)
Atlanta criteria of severity, *n* (%)	
Mild	261 (63.7)
Moderate	104 (25.8)
Severe	45 (11)
Pancreatic necrosis	
Intrapancreatic necrosis	77 (18.8)
Extrapancreatic necrosis	97 (23.7)
Scores and laboratory tests at admission	
APACHE-II, mean ± SD	6.7 ± 3.6
BISAP score, mean ± SD	1.5 ± 1.2
Haematocrit (%), mean ± SD	42.4 ± 5.3
C-reactive protein (mg/dl), mean ± SD	5.2 ± 7.8
BUN (mg/dl), mean ± SD	20.8 ± 11.1
Outcomes	
Persistent organ failure	49 (12)
Persistent multiorgan failure	31 (7.6)
Infected pancreatic necrosis	27 (6.6)
Mortality	23 (5.6)

APACHE-II, Acute Physiology and Chronic Health Evaluation-II; BUN, blood urea nitrogen; CRP, C-reactive protein; BMI, body mass index; ERCP, endoscopic retrograde cholangiopancreatography; ASA, American Society of Anesthesiologist.

**Table 2:** Outcomes of AP according BISAP, APACHE-II, BUN, and haematocrit.

	Rise in haematocrit at 24 hours, variation >0.6%				Rise in BUN at 24 hours, variation >1.87 mg/dl			
	Positive, *n* = 36	Negative, *n* = 374	*p*	OR	Positive, *n* = 65	Negative, *n* = 345	*p*	OR
Mortality, *n* (%)	8 (22.2%)	15 (4%)	0.000	6.8 (2.6–17.5)	18 (27.7%)	5 (1.4%)	0.000	26.0 (9.2–73.4)
Persistent multiorgan failure, *n* (%)	11 (30.6%)	20 (5.3%)	0.000	7.7 (3.3–18.0)	22 (33.8%)	9 (2.6%)	0.000	19.1 (8.2–44.1)
Severe AP, *n* (%)	11 (30.6%)	34 (9.1%)	0.001	4.4 (1.9–9.7)	25 (38.5%)	20 (5.8%)	0.000	10.1 (5.1–19.9)
	APACHE-II > 8				BISAP > 2			
	Positive, *n* = 120	Negative, *n* = 290	*p*	OR	Positive, *n* = 184	Negative, *n* = 226	*p*	OR
Mortality, *n* (%)	16 (13.3%)	7 (2.4%)	0.000	6.2 (2.4–15.5)	22 (12%)	1 (0.4%)	0.000	30.5 (4.0–228.9)
Persistent multiorgan failure, *n* (%)	21 (17.5%)	10 (3.4%)	0.000	5.9 (2.7–13.0)	30 (16.3%)	1 (0.4%)	0.000	43.8 (5.9–324.6)
Severe AP, *n* (%)	29 (24.2%)	16 (5.5%)	0.000	5.4 (2.8–10.5)	44 (23.9%)	1 (0.4%)	0.000	70 (9.6–519.0)
	Haematocrit at admission > 45%				BUN at admission >23.3 mg/dl			
	Positive, *n* = 125	Negative, *n* = 285	*p*	OR	Positive, *n* = 124	Negative, *n* = 286	*p*	OR
Mortality, *n* (%)	14 (11.2%)	9 (3.2%)	0.001	3.8 (1.6–9.1)	15 (12.1%)	8 (2.8%)	0.000	4.7 (1.9–11.6)
Persistent multiorgan failure, *n* (%)	20 (16%)	11 (3.9%)	0.000	4.7 (2.1–10.2)	20 (16.1%)	11 (3.8%)	0.000	4.8 (2.2–10.3)
Severe AP, *n* (%)	24 (19.2%)	21 (7.4%)	0.000	2.9 (1.5–5.6)	33 (26.6%)	12 (4.2%)	0.000	8.2 (4.1–16.7)

BISAP, Bedside Index for Severity in Acute Pancreatitis; APACHE-II, Acute Physiology and Chronic Health Evaluation-II; BUN, blood urea nitrogen; AP, acute pancreatitis.

**Table 3:** Sensitivity, specificity, and AUC values of BISAP, APACHE-II, BUN, and haematocrit regarding mortality, persisting multiorgan failure, and severe AP.

	Sensitivity (CI 95%)	Specificity (CI 95%)	AUC (CI 95%)	*p*
Mortality				
Rise in haematocrit at 24 h	47.83% (26.8%–69.4%)	85.53% (81.6%–88.9%)	0.674 (0.626–0.719)	0.0135
Rise in BUN at 24 h	78.26% (56.3%–2.5%)	87.60% (83.9%–90.7%)	0.842 (0803–0.875)	0.0001
APACHE-II	69.57% (47.1%–86.8%)	73.13% (68.4%–77.5%)	0.756 (0.711–0.797)	<0.0001
Haematocrit at admission	60.87% (38.5%–80.3%)	71.32% (66.5%–75.8%)	0593 (0.544–0.641)	0.223
BUN at admission	65.22% (42.7%–883.6%)	71.83% (67.1%–76.3%)	0698 (0.651–0.742)	<0.0001
BISAP	95.65% (78.1–99.9)	58.14 (53.0–63.1)	0.836 (0.796–0.870)	<0.0001
Multiorgan persistent failure				
Rise in haematocrit at 24 h	48.39% (30.2%–66.9%)	86.28% (82.4%–89.6%)	0.678 (0.630–0.723)	0.0038
Rise in BUN at 24 h	70.97% (52%–85.8%)	88.65% (85.0%–91.7%)	0.828 (0.787–0.863)	<0.0001
APACHE-II	67.74% (48.6%–83.3%)	73.88% (69.1%–78.2%)	0.741 (0.695–0.782)	<0.0001
Admission haematocrit	67.74% (48.6%–83.3%)	69.66% 64.8%–74.2%)	0683 (0.635–0.728)	0.0019
BUN at admission	70.97% (52.0%–85.8%)	68.34% (63.4%–73.0%)	0.721 (0.675–0.764)	<0.0001
BISAP	96.77 (83.3–99.9)	59.37 (54.2–64.4)	0.850 (0.811–0.883)	<0.0001
Atlanta severe AP				
Rise in haematocrit at 24 h	33.33% (20.0%49.0%)	85.75% (81.7%–89.2%)	0.587 (0.538–0.635)	0.0779
Rise in BUN at 24 h	55.56% (40.0%–70.4%)	89.04% (89.04%–92.1%)	0.756 (0.711–0.797)	<0.0001
APACHE-II	82.22% (67.9%–92.0%)	63.29% (58.1%–68.2%)	0.761 (0.717–0801)	<0.0001
Haematocrit at admission	57.78% (42.2%–72.3%)	69.86% (64.9%–74.5%)	0.633 (0.584–0.680)	0.0073
BUN at admission	75.56% (60.5%–87.1%)	73.79% (68.9%–78.1%)	0.787 (0.744–0.825)	<0.0001
BISAP	97.78% (88.2%–99.9%)	61.64% (56.4%–66.7%)	0.873 (0.837–0.904)	<0.0001

BISAP, Bedside Index for Severity in Acute Pancreatitis; APACHE-II, Acute Physiology and Chronic Health Evaluation-II; BUN, blood urea nitrogen; CRP, C-reactive protein, AUC, area-under-the-curve; CI, confidence interval.

## Data Availability

The data used to support the findings of this study are restricted by the Vall d'Hebrón Ethical Committee Board in order to protect patient privacy. The data are available from Vall d'Hebron Institute of Research, Edifici Mediterrània Hospital Universitari Vall d'Hebron, Passeig de la Vall d'Hebron, 119–129, 08035 Barcelona, for researchers who meet the criteria for access to confidential data.

## Abbreviations

BUN: Blood urea nitrogen
APACHE-II: Acute Physiology and Chronic Health Evaluation-II
BISAP: Bedside Index for Severity in Acute Pancreatitis.
